# Essential tremor plus rest tremor: current concepts and controversies

**DOI:** 10.1007/s00702-022-02516-2

**Published:** 2022-06-07

**Authors:** Roberto Erro, Cristiano Sorrentino, Maria Russo, Paolo Barone

**Affiliations:** 1 Department of Medicine, Surgery and Dentistry “Scuola Medica Salernitana”, Neuroscience Section, University of Salerno, Via Allende, Baronissi, SA Italy; 2 University Hospital “San Giovanni di Dio e Ruggi d’Aragona”, Salerno, Italy

**Keywords:** Aging-related tremor, Dystonic tremor, Lewy body pathology, Cerebellar pathology, Functional MRI

## Abstract

Since the initial description of Essential Tremor (ET), the entity of ET with rest tremor has proven to be a controversial concept. Some authors argued it could be a late manifestation of ET, others suggested it could be a variant of ET, yet others suggested it could represent a transitional state between ET and Parkinson's disease. The novel tremor classification has proposed the construct of ET-plus to differentiate patients with rest tremor from pure ET. However, there is no clarity of what ET-plus rest tremor represents. With the aim of shedding light on this controversial entity, we have, therefore, systematically reviewed all clinical, electrophysiological, imaging and anatomopathological studies indexed in the Medline database published both before and after the new tremor classification and involving patients with ET-plus rest tremor. Forty-four studies involving 4028 patients were included in this review and analyzed in detail by means of descriptive statistics. The results of the current review suggest that ET-plus rest tremor is a heterogenous group of conditions: thus, rest tremor might represent a late feature of ET, might reflect a different disorder with higher age at onset and lower dependance on genetic susceptibility than ET, might suggest the development of Parkinson's disease or might indicate a misdiagnosis of ET. The reviewed lines of evidence refuse recent claims arguing against the construct of ET-plus, which should be viewed as a syndrome with different possible underpinnings, and highlights methodological issues to be solved in future research.

The concept that some patients with Essential Tremor (ET) might also exhibit rest tremor (RT) was already acknowledged in the Macdonald Critchley's seminal paper titled “Observations on essential (heredofamilial) tremor” published in 1949 (Critchley 1949). Charles D. Marsden later adapted Critchley's proposal into his clinical classification of the variants of ET and also acknowledged that RT could be observed in severe ET cases (ie, ET type 3) (Marsden et al. 1984). However, the entity of ET with RT has since proven to be a controversial concept (Quinn et al. 2011; Espay et al. 2017) with different hypotheses put forward: some argued it could reflect a more advanced stage of ET (Louis 2021; Cohen et al. 2003), while others suggested it being a transitional state between ET and Parkinson's Disease (PD) (Bellows and Jankovic 2022); yet, another possibility is that it represents a different entity altogether (Rapoport et al. 1990), as presumed by the recently released tremor classification (Bhatia et al. 2018). Facing this uncertainty, the new tremor classification by the International Parkinson's and Movement Disorder Society (IPMDS) has revised the nosology of tremor syndromes and created the diagnostic category of ET-plus for those patients fulfilling the criteria for ET, but also exhibiting RT (or additional “soft signs” that do not suffice to make an alternative diagnosis) (Bhatia et al. 2018). This direction represents a notable departure from prior descriptions of ET and owes to the lack of consensus among the panel of experts on which additional signs were acceptable within the definition of ET; yet, this proposition does not admittedly seem to be based on empirical evidence.

Therefore, in this work we systematically reviewed available literature published both before and after the new IPMDS classification on tremor to collate different lines of evidence and bring clarity on the entity of ET-plus RT (ET + RT). Such an effort further aims identifying unsolved questions to guide future research, also in view of the renewed interest in tremor research boosted by the new tremor classification and the enlightened debate that has followed.

## Search strategy

In February 2022, we searched the Medline database (via PubMed, a service of the National Library of Medicines National Center for Biotechnology Information; http://www.ncbi.nlm.nih.gov) using the following term combinations: “Essential tremor” AND “rest*”, and “Essential Tremor plus”. For the last search we applied a date filter (ie, 04/06/2017, date of the formal acceptance of the IPMDS tremor classification (Bhatia et al. 2018)). After exclusion of duplicated articles, titles and abstracts were reviewed and when appropriate from the standpoint of the aim of the current review, articles were shortlisted and reviewed in detail by two reviewers (CR, MR), any disagreement being solved with the involvement of a third reviewer (RE). Based on the full text, we selected original research articles reporting prevalence, demographic, clinical or otherwise features of ET + RT with respect of ET. We excluded articles where details about ET + RT were not provided (ie, in which author reported data about ET in general, at group level) and those dealing with the “overlap” of ET and PD (ie, including patients with a former diagnosis of ET that were found to have signs of PD at the time of evaluation) given the impossibility of extracting reliable data about the entity of ET + RT and further because this particular topic has been already covered in the literature (Algarni and Fasano 2018). We further excluded articles involving patients with isolated RT in the absence of action tremor, as these patients cannot be diagnosed with ET according to the new tremor classification (Bhatia et al. 2018). Only articles published in English were included. The reference lists of the retrieved articles were also checked to include relevant papers that were missed or were not indexed in the electronic database. This systematic review adhered to the suggested guidelines for systematic reviews of observational studies (Mueller et al. 2018).

## Results

The search strategy yielded a total of 665 results, of which 44 involving a total of 4028 patients were selected for the current review. These were divided in three categories, namely studies from which demographics and clinical features could be gathered, studies using imaging or electrophysiological techniques and anatomopathological studies, as continued below.

### Clinical studies

Demographics and clinical features of ET + RT have been extracted from 23 studies involving 3500 patients. A summary of these studies is provided in Table 1. Prevalence of ET + RT ranged from as low as 1.9% (Louis et al. 2015) to 72.9% of ET cases (Rajalingam et al. 2018). Importantly, the prevalence of RT in ET has reported to be dependent on the recruitment setting, being the lowest in population-based setting and highest in a brain bank study (Louis et al. 2015). In many (Rajalingam et al. 2018; Bellows and Jankovic 2021; Campbell et al. 2022; Prasad and Pal 2019; Peng et al. 2022; Gilmour et al. 2021; Steffen et al. 2020), but not all studies (Pandey and Bhattad 2021; Manorenj et al. 2019), ET + RT was among the commonest ET-plus subtypes.

**Table 1:** Summary of clinical studies on plus rest tremor

First author, year	Sample	Main findings	Conclusions
Koller, 1985	8 patients with “combined resting-postural” tremor	Patients has mean age of 60 years and a mean disease duration of 10.6 years. Two patients had a positive FH of ET, and one of PD. The postural component, which was not re-emergent, had a higher amplitude than the resting component. In all cases it started unilaterally, but became bilateral, yet asymmetric. None had bradykinesia or rigidity. Anti-tremor drugs (trihexyphendinil and propranolol) as well as l-dopa failed to reduce tremor, but one patient reported benefit from alcohol	The authors support the existence of various ET “subtypes” (RT can be observed either in severely affected or elderly patients)
Martinelli, 1987	200 consecutive patients with ET	Out of 200 ET cases, 25 (12.5%) had rest tremor, none of whom had bradykinesia or rigidity. In 19 patients rest and postural tremors were present since onset, while in 4 patients it started as postural tremor and 1 as resting tremor, in all 5 patients becoming continuous in about 1 year. These patients had a higher age at onset (55 years) than patients with “typical” ET (50 years) and FH of tremor was less common (28% vs 43%). No patients developed PD during the follow-up of 2–5 years	ET + RT represent a different ET “subtype” with higher age at onset
Cohen, 2003	64 patients with ET	Out of 64 ET cases, 12 (18.75%) had rest tremor. None of these had signs of parkinsonism. These had a longer disease duration than ET without rest tremor, their tremor more commonly involved the head (50% vs 21%) and the kinetic/postural tremor was more severe. Rest tremor was bilateral in 67% of these cases. A logistic regression showed that disease duration was the only variable associated with rest tremor	RT might develop as a function of longer ET duration
Louis, 2003	71 patients with ET, whose smell ability was evaluated with the University of Pennsylvania Smell Identification Test (UPSIT)	Out of 71 ET cases, 13 (18.3%) had rest tremor. These were older than ET cases without rest tremor (73.7 vs 66.6 years), but had a similar disease duration of about 22–24 years. Their total tremor score was higher than ET case without rest tremor. UPSIT score did not differ between the two groups	ET + RT represent a different ET “subtype” with higher age at onset. These patients have normal smell performances which would exclude PD
Louis, 2005	94 patients with ET	Out of 94 ET cases, 31 (32.9%) had rest tremor These patients were older than patients without rest tremor (78 vs 72 years), and had higher mean total tremor score, despite a similar mean disease duration of about 40 years. Cranial-cervical involvement was similar between the two groups	ET + RT represent a different ET “subtype” with higher age at onset
Djaldetti, 2008	24 patients with ET who were evaluated with the UPSIT and compared to 17 PD patients and 9 healthy subjects	Out of 24 ET cases, 7 (29.16%) had rest tremor. These were older and had a longer disease duration than ET cases without rest tremor There was no significant difference in the UPSIT score between the two groups, nor between them and healthy subjects, whereas PD cases has significantly lower scores	ET + RT represent a different ET “subtype” with higher age at onset, but also longer ET duration (RT can be observed either in severely affected or elderly patients). These patients have normal smell performances which would exclude PD
Uchida, 2011	11 patients with ET + RT and 38 with PD	The intensity of rest tremor was markedly attenuated during walking relative to resting in ET + RT, as opposed to PD	The phenomenological features of ET + RT would exclude PD
Louis, 2012	55 patients with ET who were tested for color vision abnormalities by means of the Farnsworth–Munsell 100 Hue test	Out of 55 ET cases, 7 (12.72%) had rest tremor and were similar with respect to age, gender, education, MMSE score and tremor duration to ET cases without rest tremor. No differences were found between the two groups in terms of color discrimination performance	ET + RT has normal color discrimination performances which should exclude PD
Martinez-Hernandez, 2014	100 patients with ET whose writing was analyzed in terms of height and width of the most commonly repeated letters	Out of 100 ET cases, 15 (15%) has rest tremor. Writing analysis did not show any significant differences between the two groups (i.e., none showed micrographia)	ET + RT had no micrographia which would exclude PD
Louis, 2015	831 patients with ET recruited in four distinct settings (population, genetics study, study of environmental epidemiology, brain bank)	The prevalence of rest tremor was lowest in the population-based setting (1.9%), highest in the brain bank study (46.4%), and intermediate in the remaining two settings (9.6% and 14.7%). Rest tremor was associated with older age and greater tremor severity. Only in one of three examined setting, ET cases with rest tremor had longer disease duration than ET cases without. They were more likely to have voice tremor than ET cases without rest tremor	The prevalence of RT in ET depends on the type of recruitment. ET + RT represent a different ET “subtype” with higher age at onset, and more severe tremor (RT can be observed either in severely affected or elderly patients)
Rajalingam, 2018	133 patients with lower limb involvement formerly diagnosed as ET	Out of 133 ET cases, 97 (72.9%) had rest tremor. These patients were older (71.5 vs 52.3 years) and had longer disease duration (36.8 vs 26.1 years) than ET without rest tremor, but has a similar age at onset (34.8 vs 26.2 years)	RT might develop as a function of longer ET duration
Manorenj, 2019	45 patients with ET	Out of 45 ET cases, 6 (13.3%) had rest tremor	RT in ET is not very frequent
Prasad, 2019	183 patients with a former diagnosis of ET	Out of 183 cases, 62 (33.8%) had rest tremor. ET + RT was the commonest ET-plus subtype	ET + RT is the commonest ET-plus subtype
Bugalho, 2020	43 patients with ET/ET-plus, who were screened for RBD using the RBDSQ and video-polysomnography	Out of 43 cases, 18 (41.86%) had rest tremor. Overall, RBD was confirmed in 5 cases. Rest tremor was present in 100% of these cases and in 34.2% of cases without RBD (p < 0.001)	RT is much commoner in ET patients with confirmed RBD, suggesting superimposed PD
Steffen, 2020	44 patients with a former diagnosis of ET	Out of 44 cases, 14 (31.1%) had rest tremor. ET + RT was the commonest ET-plus subtype	ET + RT is the commonest ET-plus subtype
Bellows,2021	300 patients with a former diagnosis of ET	Out of 300 formerly diagnosed cases, 160 (53.3%) were reclassified as ET-plus, of whom 64 (40%) had rest tremor	ET + RT is among the commonest ET-plus subtypes
Gilmour, 2021	26 patients with a former diagnosis of ET	Out of 26 cases, 4 (15.4%) had rest tremor. ET + RT was the second commonest ET-plus subtype. after ET-plus questionable dystonia	ET + RT is among the commonest ET-plus subtypes
Iglesias-Hernandez, 2021	201 patients with ET/ET-plus followed up for up to 64 months	The proportion with ET plus increased from 58.7% at baseline to 72.1%. Of 172 (85.6%) who received a diagnosis of ET-plus during the observation period, reversion to ET was observed in 62 (36.0%).Rest tremor was the most unstable clinical feature: it was present in 59 subjects (29.3% of the entire cohort), among whom it reverted from present to absent in 38.9%	RT is an unstable feature which might disappear at follow-up
Louis, 2021	241 patients formerly diagnosed with ET	Up to 28.7% of patients had rest tremor (28.7% while seated vs 8.4% while standing). In younger cases (onset < 40 years), rest tremor correlated with age and disease duration, whereas in older cases it correlated with age but not the disease duration	ET + RT is heterogeneous. RT correlated with disease duration only in young-onset cases, whereas it correlated with age only in old-onset cases
Pandley, 2021	45 prospectively assessed patients with ET-plus	Out of 45 ET-plus cases, only 2 (4.4%) had rest tremor	RT in ET is not very frequent
Campbell, 2022	92 patients with a former diagnosis of ET assessed for deep brain stimulation or focused ultrasound thalamotomy	87% of these patients were reclassified as ET-plus. ET + RT was the commonest ET-plus subtype (70.2%)	ET + RT is the commonest ET-plus subtype
Nisticò, 2022	24 patient with ET + RT and 90 patients with PD	Rest tremor was observed in all cases in both groups, in the hand-hanging position, whereas it was more commonly observed in PD in the standing condition (75.6% vs 45.8%) and when lying down (67.8% vs 37.5%)	RT prevalence is influenced by the position of the arms during tremor assessment
Peng, 2022	665 patients with a former diagnosis of ET of those 274 were diagnosed by ET, 391 were ET plus, the most prevalent soft sign was rest tremor (66%)	Out of 665 cases, 391 (58.7%) were reclassified as ET-plus. Rest tremor was the commonest soft sign (present in 66% of ET-plus cases) and, along with questionable cerebellar signs, was associated with tremor severity. In general, ET-plus cases were older, had higher age at onset and longer disease duration than ET cases	ET + RT is the commonest ET-plus subtype

*ET* Essential Tremor, *ET* + *RT* Essential Tremor with Rest Tremor, *FH* Family History, *MMSE* Mini-mental state examination, *PD* Parkinsons Disease, *RBD* REM Sleep Behaviour Disorder, *RBDSQ* RBD Screening Questionnaire, *UPSIT* University of Pennsylvania Smell Identification Test

In the vast majority of studies reporting this information, patients with ET + RT were older than ET (Louis et al. 2015, 2005; Rajalingam et al. 2018; Campbell et al. 2022; Peng et al. 2022; Louis and Jurewicz 2003; Djaldetti et al. 2008), with only one study reporting a similar age between ET + RT and ET (Louis et al. 2012). There are conflicting evidence of whether the latter result depends on longer disease duration of ET + RT with respect of ET without RT. Four studies found a longer disease duration in ET + RT than in ET (Cohen et al. 2003; Rajalingam et al. 2018; Prasad and Pal 2019; Djaldetti et al. 2008), whereas three did not (Louis and Jurewicz 2003; Louis et al. 2005, 2012). One study (Louis et al. 2015) found this relationship to be true only in one of four examined settings (ie, population-based, genetic study, study of environmental epidemiology, brain bank) and another study found this association only in young onset cases (< 40 years) but not in patients with older age at onset (Louis et al. 2021). In keeping with the latter finding, the majority of the studies (Louis et al. 2015, 2005; Peng et al. 2022; Louis and Jurewicz 2003; Martinelli et al. 1987), but not all (Rajalingam et al. 2018; Djaldetti et al. 2008; Louis et al. 2012), reported patients with ET + RT having a higher age at onset than ET. Many studies found ET + RT to be more severely affected than ET cases (Louis et al. 2015; Peng et al. 2022; Louis and Jurewicz 2003) and to have more likely cranial-cervical tremor (Cohen et al. 2003; Louis et al. 2015), although this finding was not confirmed in another study (Louis et al. 2005).

Although the majority of studies did not detail the onset of RT with regard to action tremor, it was commonly reported as a late feature of the condition. However, the only study which detailed these data reported 76.3% of cases having action tremor and RT since onset, 16.6% developing action tremor first and 4.1% developing RT first (Martinelli et al. 1987).

The majority of the clinical studies did not detail the distribution of RT in ET + RT and it has been reported to be either unilateral or bilateral (either symmetric or asymmetric) (Cohen et al. 2003; Koller and Rubino 1985). RT in ET + RT has been reported to be not re-emergent upon holding a posture (Koller and Rubino 1985; Papengut et al. 2013), to be significantly attenuated during walking (Uchida et al. 2011) and to be less common during standing (Louis et al. 2021; Nisticò et al. 2022) and when lying down as compared to PD (Nisticò et al. 2022). The other studies comparing ET + RT to ET (without RT) for features suggestive of PD found no differences between the two groups in terms of smell performances (Louis and Jurewicz 2003; Djaldetti et al. 2008), writing (Martinez-Hernandez and Louis 2014) and color discrimination (Louis et al. 2012). However, another study found REM sleep Behavior Disorder (RBD) in 11.6% of ET cases and RT to be more common in ET with RBD (100%) as compared to ET without RBD (34.2%) (Bugalho et al. 2021).

Interestingly, the available longitudinal studies showed that no patients with ET + RT developed PD during a follow-up of 24–64 months (Gilmour et al. 2021; Martinelli et al. 1987; Bugalho et al. 2021), but also that RT was an unstable feature, reverting from present to absent in more than one-third of cases (Iglesias-Hernandez et al. 2021).

Overall, the majority of the studies reported patients with ET + RT to have higher age at onset and a greater tremor severity than ET. Moreover, these patients missed clinical features suggestive of parkinsonism.

### Electrophysiological and imaging studies

Six studies involving a total of 60 patients employed electrophysiology in ET + RT. Thus, RT in ET-plus cases has been shown to have a synchronous EMG pattern as opposed to the alternating pattern observed in PD (Fekete and Li 2013; Nisticò et al. 2011; Vescio et al. 2021), a finding that has been suggested to differentiate the two conditions at individual level. Conversely, tremor frequency measurement either at rest or across different activating conditions does not distinguish the two conditions (Cichaczewski et al. 2014). Interestingly, Nisticò et al. explored the integrity of the blink reflex recovery cycle (BRRC) in ET + RT and ET, showing that the former had an increased R2 component of the BRRC (Nisticò et al. 2012). Given that these patients had a normal DAT-SPECT, they suggested ET + RT to be dystonic in nature rather than being an ET subtype (Nisticò et al. 2012). On the other hand, another study reported patients with ET + RT to have electrophysiological features suggestive of parkinsonism (ie, slow spiral drawing speed and increased decrement of spiral drawing speed with radius) (Cohen et al. 2003). Overall, the electrophysiological features of ET + RT seem to be similar to those of ET. Yet, individual studies reported electrophysiological features consistent with dystonia or suggestive of parkinsonism.

The hypothesis that ET + RT might be sustained by a dopaminergic denervation has been tested in 10 studies using DAT-SPECT and involving a total of 192 patients. Thus, Lee et al. reported patients with ET + RT to have intermediate uptake binding values (suggesting a mild substantia nigra neuronal loss) between ET without RT and patients with PD (Lee et al. 1999). In these patients the mean disease duration was of about 18 years (but RT only developed as a late feature for a mean duration of approximately 3 years) as opposed to 16 years of patients with ET without RT (Lee et al. 1999). In the aforementioned study by Djaldetti et al. (Djaldetti et al. 2008), 25% of ET + RT cases (mean disease duration of about 15 years) had abnormal DAT-SPECT findings despite normal smell performances. A similar frequency was found in another study in which 22% of patients with ET + RT (mean disease duration of about 11 years) had evidence of dopaminergic denervation (You et al. 2013), whereas a much higher figure has been found in other three studies (Ceravolo et al. 2008; Verdal et al. 2013; Marsala et al. 2017). Thus, Ceravolo et al. reported 50% of patients with ET + RT (mean disease duration of about 10 years) to have abnormal DAT-SPECT (Ceravolo et al. 2008). Interestingly, 60% of these had a clinical evolution to PD, whereas the remaining 40% still presented with an isolated tremor syndrome at the follow-up of about 2 years (Ceravolo et al. 2008). de Verdal et al. reported 75.7% of patients with ET + RT (mean disease duration of 13 years) with an abnormal DAT-SPECT, of whom about the half had unilateral RT and the other half bilateral RT (Verdal et al. 2013). Finally, Marsala et al. found 88.2% of abnormal scans in patients with ET + RT with a mean disease duration of about 19 years (Marsala et al. 2017). At a mean follow-up of 5 years, these patients showed mild parkinsonian features, such as the reduction of pendular movements, micrography, and slight muscle rigidity (Marsala et al. 2017). In contrast to the above, Asenbaum et al. found normal DAT-SPECT findings in all 32 investigated patients with ET, of whom 18 (56.25%) had RT (Asenbaum et al. 1998). Of note, in this study patients with ET + RT had a shorter disease duration (of about 9.3 years) than ET patients without RT (11.5 years) (Asenbaum et al. 1998). Similar findings were obtained in three more studies (Fekete and Li 2013; Nisticò et al. 2012; Caligiuri et al. 2017) in which all patients with ET + RT had normal SPECT findings. Therefore, the percentage of abnormal DAT-SPECT findings grossly varied across studies and ranges from 0% to 88.2% of the recruited sample, with higher figures being reported in patients with longer disease duration.

Seven studies involving a total of 265 patients used different magnetic resonance imaging (MRI) techniques to explore the neuroanatomical correlates of ET + RT (Caligiuri et al. 2017; Nicoletti et al. 2015; Li et al. 2021, 2020; Novellino et al. 2016; Prasad et al. 2018; Cherubini et al. 2014). A connectivity-based study by Caliguri et al. found common alterations of the cerebellothalamo-cortical (CTC) network in ET + RT and ET cases without RT, with the former additionally showing reduced connectivity in a pathway connecting globus pallidus, caudate, and supplementary motor area (Caligiuri et al. 2017). In keeping with these results, in a task-based (ie, during continuous writing) functional MRI (fMRI) Nicoletti et al. found a reduced activation of various cortical areas and internal globus pallidus in ET + RT, beyond the shared reduced activation of the CTC network in both ET and ET + RT (Nicoletti et al. 2015). They additionally failed to evidence morphometric changes between the two groups (Nicoletti et al. 2015). As opposed to the above, Li et al. explored spontaneous brain activity in a resting-state fMRI in ET and ET + RT showing that two groups shared a reduced activity in the basal ganglia, but differed in terms of cerebellar and limbic system activity, respectively (Li et al. 2021). Novellino et al. explored cerebellar microstructure in patients with ET + RT as compared to ET cases without RT and healthy controls (HC) by Diffusion Tensor Imaging (DTI) (Novellino et al. 2016). Thus, whereas all patients as a group showed increased cerebellar gray matter (GM) diffusivity than HC, ET + RT showed intermediate values between ET without RT and HC, suggesting that cerebellar involvement does not seem to fully account for ET + RT, with additional networks being possibly involved (Novellino et al. 2016). In contrast with these results, however, Prasad et al. (Prasad et al. 2018) found no abnormalities in cerebellar GM between ET + RT and ET without RT, nor when comparing these groups to HC, in keeping with results obtained with voxel-based morphometry (Nicoletti et al. 2015). However, they found a consistent pattern suggestive of higher cerebellar white matter (WM) microstructural damage in ET + RT, suggesting this could be a severe variant of ET (Prasad et al. 2018). Of note and differently from Novellino (Novellino et al. 2016), Prasad et al. adopted a whole-brain approach and did not confirm basal ganglia involvement in ET + RT (Prasad et al. 2018). Two studies compared ET + RT with tremulous PD (Li et al. 2020; Cherubini et al. 2014). Thus, Li et al. using a resting-state fMRI showed a decreased activation of the default mode network, bilateral putamen and bilateral cerebellum in both groups, with patients with PD additionally showing alterations in the bilateral supplementary motor area and precentral gyrus (Li et al. 2020). Finally, Cherubini et al. used a support vector machine (SVM) approach combining morphometric GM and WM data with DTI-derived mean diffusivity and fractional anisotropy data to distinguish ET + RT from PD (Cherubini et al. 2014). They showed that SVM classification of individual patients was not accurate if based on a single predictor, whereas when all predictors were combined in a multimodal algorithm, SVM distinguished patients with ET + RT from PD cases with an accuracy of 100% (Cherubini et al. 2014). However, this study did not provide any information on the specific abnormalities observed in each group, therefore hampering any speculations on the involved circuitry in the generation of RT in either disorder. Overall, the seven studies exploiting MRI did not show consistent findings and only suggested that the abnormality responsible for RT might localize in a circuitry different from that causing action tremor.

### Anatomopathological studies

Only 2 studies involving a total of 38 patients explored post-mortem findings in ET + RT. Thus, in a series of 20 cases with ET, of whom 6 had additional RT, Rajput et al. found no consistent pathology at a group level, nor differences between ET + RT and ET (Rajput et al. 2004). Similarly, Louis et al. (Louis et al. 2011) compared 9 cases with ET + RT with 9 age-matched cases with ET and found no differences between the two groups. Namely, in both there was found an increased number of torpedoes of the cerebellar cortex and lower Purkinje cells, as compared to normative control values, whereas neither demonstrated Lewy body-containing neurons, Lewy neurites or other relevant pathological changes in the basal ganglia were minimal, suggesting that ET + RT is not the expression of underlying Lewy body pathology (Louis et al. 2011). Overall, these two anatomopathological studies did not demonstrate PD pathology in ET + RT.

## Discussion

**Fig. 1:**
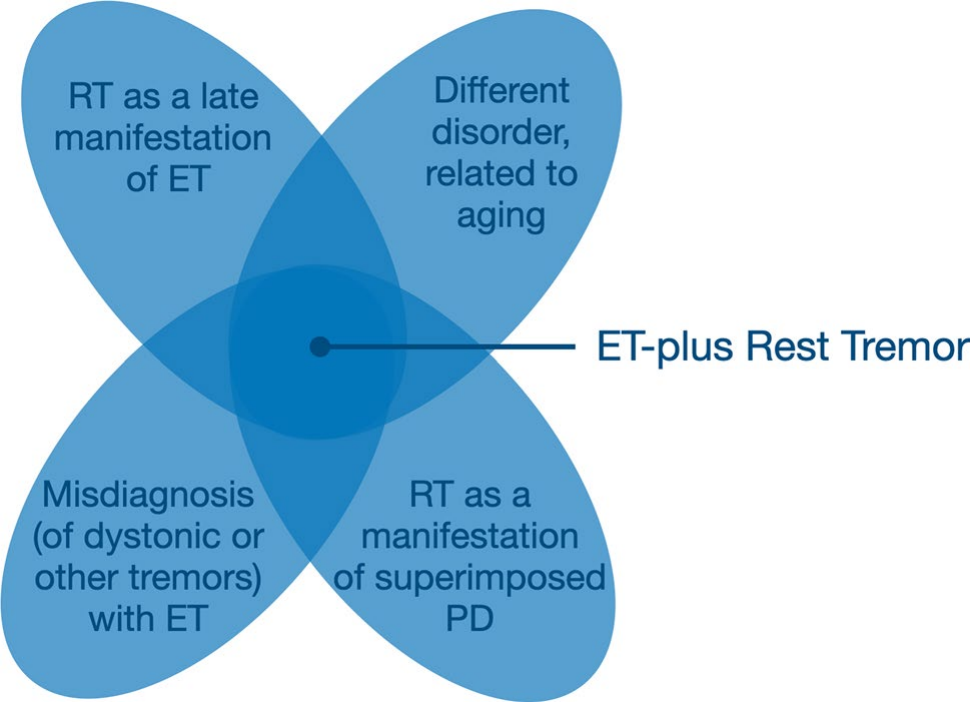
The heterogeneous underpinnings of ET-plus rest tremor. All conditions result in the presence of rest tremor in addition to action tremor, but other clinical features might also be shared between these conditions loosening their boundaries in patients at the borders of their respective classification. *ET* Essential Tremor, *PD* Parkinsons Disease, *RT* Rest Tremor

The entity of ET + RT is relatively common: RT has been reported to occur in up to 72.9% of ET cases (Rajalingam et al. 2018), although its reported prevalence might depend on the specific research setting (Louis et al. 2015), and ET-plus RT is among the commonest ET-plus subtypes in many studies (Rajalingam et al. 2018; Campbell et al. 2022; Prasad and Pal 2019; Peng et al. 2022; Gilmour et al. 2021; Steffen et al. 2020; Louis et al. 2021). However, the clinical, electrophysiological, imaging and anatomopathological lines of evidence reviewed above clearly indicate that ET + RT is a heterogenous entity (Fig. 1), which is in keeping with the current conceptualization of ET-plus being a syndrome and not a single disease (Bhatia et al. 2018). The conclusions that would be gleaned, and will be discussed in turn, might be that: (1) RT might develop as a relatively late feature in long-standing ET cases; (2) ET + RT represents a different entity from ET, arguably sustained by different pathophysiological mechanisms; (3) the development of RT in ET cases might indicate a superimposed diagnosis of PD; and (4) these patients might have a different disorder, such as dystonic tremor, misdiagnosed as ET.

### Rest tremor as a late manifestation of essential tremor

There is a body of works suggesting that RT can be a late manifestation of ET (Cohen et al. 2003; Louis et al. 2015, 2021; Rajalingam et al. 2018; Djaldetti et al. 2008), which echoes the initial suggestions by Critchley (Critchley 1949) and Marsden (Marsden et al. 1984). In keeping with this hypothesis, patients with ET + RT had longer disease duration and greater tremor severity than ET cases without RT. Supporting this view and excluding the possibility that these cases might have developed PD, there are also studies showing preserved dopaminergic functionality, as assessed by DAT-SPECT in the majority of these patients (Djaldetti et al. 2008; Fekete and Li 2013; Nisticò et al. 2012; Lee et al. 1999; You et al. 2013; Asenbaum et al. 1998; Caligiuri et al. 2017) as well as anatomopathological studies failing to demonstrate Lewy body pathology (Rajput et al. 2004; Louis et al. 2011). Moreover, the electrophysiological studies comparing ET + RT and PD cases further demonstrated tremor characteristics (i.e., synchronous pattern) in the former that bear resemblance to those of action tremor in ET and contrast to what is observed in parkinsonian tremor (Fekete and Li 2013; Nisticò et al. 2011; Vescio et al. 2021). The evidence that RT might arise from the involvement of a different circuitry of that sustaining action tremor (Caligiuri et al. 2017; Nicoletti et al. 2015; Li et al. 2021; Novellino et al. 2016) does not have a unique interpretations: following the account that RT develops as a late manifestation of ET, one might speculate that this phenomenon occurs as a function of the progression of the underlying disease, but alternative hypotheses are possible, as continued below.

### Essential tremor plus rest tremor reflects a different entity

Supporting an alternative hypothesis, there are evidence suggesting the RT might not reflect disease duration (and progression), but aging. Many studies reported patients with ET + RT to be older than ET cases without RT, despite a similar disease duration, and to have a higher age at onset (Louis et al. 2015, 2005, 2012, 2021; Louis and Jurewicz 2003). A proportion of these patients might therefore have tremor, including RT, as a manifestation of pathological aging (Deuschl et al. 2015), which would arguably be less dependent on genetic susceptibility (Martinelli et al. 1987). This hypothesis is intriguing further because it would recapitulate the bimodal distribution in terms of age at onset that has been classically observed in ET in general (ie, before the new tremor classification) (Haubenberger and Hallett 2018). This hypothesis has not yet been formally tested and might represent a topic for future investigation. In this context, however, it is worth noting that two studies attempted a factor analysis of the clinical features of ET (Louis et al. 2005; Louis 2009). The results from this type of approach are driven by the data and are hence not generated following a particular hypothesis (Erro et al. 2016). In both studies, RT emerged as an independent factor (Louis et al. 2005; Louis 2009), which confirms that these patients represent a different entity from ET. However, in one of the studies including age as a feature, it did not group with RT (Louis 2009), which is in contrast to the hypothesis raised above. However, it should be noted that disease duration was not included in the primary analysis, nor secondary analyses were performed to compare patients based on the identified factors to test this hypothesis. What remains from these data-driven approaches is that RT would seem to identify a group of patients different from ET. From a mechanistic standpoint, the difference between the two groups might stand in the involvement of not entirely overlapping networks, as suggested by some works using MRI (Caligiuri et al. 2017; Nicoletti et al. 2015; Li et al. 2021; Novellino et al. 2016). In such cases, the involvement of such circuitry would be therefore triggered by pathological aging rather than by the progression of the underlying disease itself.

## Rest tremor in essential tremor might indicate superimposed Parkinson's disease

Alternative lines of evidence would instead suggest the ET + RT is sustained by a dopaminergic dysfunction in up to 88.2% of cases as suggested by DAT-SPECT studies (You et al. 2013; Ceravolo et al. 2008; Verdal et al. 2013; Marsala et al. 2017), although a number of other works did not show DAT-SPECT abnormalities in these patients (Djaldetti et al. 2008; Fekete and Li 2013; Nisticò et al. 2012; Lee et al. 1999; You et al. 2013; Asenbaum et al. 1998; Caligiuri et al. 2017) and further failed to demonstrated clinical features suggestive of PD such as hyposmia (Louis and Jurewicz 2003; Djaldetti et al. 2008), micrographia (Martinez-Hernandez and Louis 2014) and reduced color discrimination (Louis et al. 2012). Although DAT-SPECT is considered as the gold standard for the evaluation of the dopaminergic presynaptic system *in-vivo*, the two available anatomopathological studies failed to reveal Lewy body pathology in ET + RT (Rajput et al. 2004; Louis et al. 2011). These conflicting results remark on the heterogeneity of patients with ET + RT and, in this context, it is worth noting that the anatomopathological features of the entity named “benign tremulous parkinsonism” (i.e., patients with RT that consistently overshadows additional non-tremor parkinsonian features, and only mild deterioration during the disease course) have also been found to be heterogenous (Selikhova et al. 2013). In a postmortem study of 21 such patients (Selikhova et al. 2013), 16 had pathologically proven PD (with relatively little nigral cell loss, thus explaining their benign phenotype), whereas 5 did not, therefore arguably falling in the category of what we would today call ET-plus. This demonstrates, from the other end of the spectrum, frequent diagnostic uncertainty in some tremulous patients at the borders of the classification of PD (Erro et al. 2016). The evidence that a number of ET + RT cases had an abnormal DAT-SPECT, despite no or minimal evidence of parkinsonism beyond RT, is supported by some imaging studies demonstrating basal ganglia involvement in these patients (Caligiuri et al. 2017; Nicoletti et al. 2015) and corroborate the admittedly controversial hypothesis that ET might evolve in PD (Bellows and Jankovic 2022, 2021). This hypothesis would be supported by the evidence that the proportion of ET + RT cases with abnormal DAT-SPECT increased in studies recruiting patients with longer disease duration. While this proposition is still under debate (Algarni and Fasano 2018), it should be noted the new tremor classification allows for the transition between diagnostic categories and does not exclude the concept of “antecedent ET” converting into a different disorder (Bhatia et al. 2018). Large longitudinal or epidemiological studies are therefore warranted to definitively confirm or dispute such hypothesis.

### Misdiagnoses of essential tremor plus rest tremor

Finally, there is also evidence that at least in a subset of patients with ET + RT the clinical diagnosis was erroneous. Nisticò et al. showed these patients having an abnormal BRRC in keeping with the diagnosis of dystonia (Nisticò et al. 2012). They subsequently suggested that this electrophysiological marker could be helpful to distinguish ET + RT from patients with PD (Nisticò et al. 2014), although the findings in the two groups largely overlapped and might not be that useful at individual level. However, the main conclusion that derives from these studies is that there might be a misdiagnosis of ET in rarer forms of tremor (Erro and Reich 2022), including dystonia. This evidence reiterates previous claims (Quinn et al. 2011) and is in line with recent findings showing that RT is common in dystonia (Erro et al. 2014). Interestingly, a recent study showed that the diagnostic allocation of tremulous patients is partly dependent on the specific expertise of the evaluators (Becktepe et al. 2021), a finding which might explain a proportion of the misdiagnoses.

## Conclusions and future directions

The body of works reviewed above indicate that ET + RT does not represent a single entity. This finding would support the newly conceptualized construct of ET-plus by the IPMDS (Bhatia et al. 2018), which is viewed as a syndrome defined by clinical features only (axis 1) and might be sustained by different etiologies (axis 2). From the standpoint of the IPMDS tremor classification (Bhatia et al. 2018), all hypotheses delineated above are permissible. One of the novelty of the classification is the acknowledgment of the possibile transition over time between different tremor syndromes (Bhatia et al. 2018). As such, a patient with ET would be labelled with ET-plus once RT has developed and similarly for those patients exhibiting ET/ET-plus who later develop PD, dystonia, or other neurological features. This concept has been clearly indicated by the IPMDS tremor classification where there is mention of “antecedent ET” transitioning into a different syndrome (Bhatia et al. 2018). On the other hand, the construct of ET-plus in itself highlights the possibility that patients with ET + RT might be different from “pure” ET. This departure from previous tremor classifications is primarily meant to recruit homogenous group of patients to guide subsequent translational research. Future longitudinal research should therefore adhere to this conceptual shift and might unravel if there are clinical or otherwise predictors of transition to other diagnostic allocations or, conversely, might demonstrate that RT is a late feature of ET in a subset of patients.

In this regard, a few methodological considerations should be done. First, it seems in truth paradoxical that RT was found to fluctuate in a longitudinal study where it reverted from present to absent in more than one-third of cases (Iglesias-Hernandez et al. 2021). This admittedly curious finding, in addition to selection biases (Louis et al. 2015), might explain the discrepancies in prevalence of RT across the reviewed studies and might be partly due to the fact that RT in ET-plus can be observed in some conditions (while seated) but not in others (while standing) (Louis et al. 2021). Moreover, different studies have shown how RT might be present/absent depending on different hand and arm positions, both in ET and PD (Nisticò et al. 2022; Wilken et al. 2019). This calls for a standardization of the evaluation of RT in tremulous patients, to ensure comparability across different studies. Second, whereas we praise research efforts using imaging techniques to study the underpinnings of ET + RT, we admit that they only provide mechanistic evidence of which circuitry might be involved (Erro et al. 2022), but essentially fail to provide any robust insights into the etiological correlates of this entity. The involvement of a specific circuitry might be shared by different disorders and, conversely, in a single disease multiple tremor types might be present as result of the involvement of different networks (Erro et al. 2022). As such, future imaging research should attempt to adopt a multi-modal approach, including the use of DAT-SPECT, which is currently viewed as the gold standard for detecting dopaminergic dysfunction and against which to corroborate (or not) the results obtained by MRI. Finally, there is a dramatic paucity of anatomopathological works on ET + RT and ET in general. A clear lesson that we have learned from pathological studies in PD is that clinical diagnostic accuracy might not be excellent, even when the diagnosis if performed by a movement disorder expert (Rizzo et al. 2016). In the era where advanced metabolic, molecular neuroimaging, genetic and “omics” techniques exist, it is now, more than ever, that postmortem brain investigations must be performed (Carlos et al. 2019). Only by comparing and integrating post-mortem with ante-mortem findings from clinical, neurophysiological, neuroimaging, and other biomarker examinations can we truly discover neurological disease mechanisms (Carlos et al. 2019).

In summary, the evidence reviewed here indicate that patients with ET + RT represent a heterogeneous group of disorders and contrast with the criticisms raised by some authors arguing against the newly conceptualized construct of ET-plus (Louis et al. 2020), who supported the idea that ET + RT, among other ET-plus subtypes, simply reflect a more advanced stage of ET (Louis et al. 2021). The suggestions we have advocated will homogenize methodology of future research efforts and bring clarity on this controversial entity.
